# Supplementary material for: Effects of Hypoxia and Reoxygenation on Hypoxia-Responsive Genes, Physiological and Biochemical Indices in Hybrid Catfish (Pelteobagrus vachelli ♀ × Leiocassis longirostris ♂)
Source: Biology (Basel). 2025 Jul 23;14(8):915. doi: 10.3390/biology14080915 (PMC12383296; doi:10.3390/biology14080915)
Supplement: Supplementary file 1 [file biology-14-00915-s001.zip › biology-3709081-supplementary.pdf]

Enzyme activity determination method

Table S1. Enzyme activity determination method

Index	Test method	Principle	Wavelength
PK	UV Colorimetry	In the presence of ADP, PK catalyzes the conversion of PEP to pyruvate, which is then converted to lactate by LDH, accompanied by the oxidation of NADH to NAD.	340 nm
HK	Spectrophotometry	HK catalyzes the conversion of glucose to glucose-6-phosphate, which is then dehydrogenated by glucose-6-phosphate dehydrogenase to produce NADPH, exhibiting a characteristic absorption peak at 340 nm.	340 nm
LDH	Colorimetry	LDH catalyzes the conversion of lactate to pyruvate, which reacts with 2,4-dinitrophenylhydrazine to form brownish-red pyruvate dinitrophenylhydrazone, measurable by colorimetry.	440 nm
CS	Spectrophotometry	CS catalyzes the reaction of acetyl-CoA and oxaloacetate to form citryl-CoA, which is hydrolyzed to citrate, converting colorless DTNB to yellow TNB with a characteristic absorbance at 412 nm.	412nm
T-SOD	WST-1 Method	WST-1 reacts with superoxide anions generated by xanthine oxidase to produce water-soluble formazan dye. SOD activity is inversely correlated with formazan dye formation, quantified by colorimetry.	450 nm
GSH-PX	Colorimetry	GSH-PX promotes the reaction of H_2O_2 with GSH to produce H_2O and GSSG. Enzyme activity is determined by measuring the consumption of GSH.	412 nm
CAT	Ammonium Molybdate	CAT catalyzes the decomposition of H_2O_2. The reaction is terminated by adding ammonium molybdate, and residual H_2O_2 forms a light-yellow complex measurable at 405 nm.	405 nm
LPO	Colorimetry	At 45° C for 60 minutes, LPO reacts with two molecules of chromogen to form a stable chromophore with maximum absorption at 586 nm. LPO content is calculated using relevant formulas.	586 nm
PC	UV Colorimetry	Carbonyl groups react with 2,4-dinitrophenylhydrazine to form red 2,4-dinitrophenylhydrazone, exhibiting a characteristic absorption peak at 370 nm.	370 nm
MDA	TBA Method	MDA, a degradation product of lipid peroxides, condenses with TBA to form a red product with maximum absorption at 532 nm.	532nm
